# Original Scientific Reports: Clinicopathological Findings of Remnant Pancreatic Cancers in Survivors Following Curative Resections of Pancreatic Cancers

**DOI:** 10.1007/s00268-015-3353-5

**Published:** 2015-11-20

**Authors:** Shuji Suzuki, Toru Furukawa, Nana Oshima, Wataru Izumo, Kyoko Shimizu, Masakazu Yamamoto

**Affiliations:** Department of Gastroenterological Surgery, Ibaraki Medical Center, Tokyo Medical University, 3-20-1 Chuo, Amimachi, Inashikigun, Ibaraki, 300-0395 Japan; Department of Gastroenterological Surgery, Tokyo Women’s Medical University, Tokyo, Japan; Institute for Integrated Medical Sciences, Tokyo Women’s Medical University, Tokyo, Japan; Department of Gastroenterology, Tokyo Women’s Medical University, Tokyo, Japan

## Abstract

**Background:**

This retrospective study aimed to evaluate clinicopathological findings of remnant pancreatic cancers in survivors of invasive ductal adenocarcinomas of the pancreas (PDAC).

**Methods:**

A group of 23 patients out of 826 who had curative resections for PDAC between 1980 and 2011 was identified and treated for metachronous pancreatic cancer.

**Results:**

The following tubular adenocarcinomas were found at the first surgery: 3 well differentiated, 17 moderately differentiated, 1 papillary, and 1 poorly differentiated. Treatments for the remnant pancreas consisted of remnant pancreatectomy in 12 patients, chemotherapy in 6, and the best supportive care in 5. The mean time to treatment was 74.2 months. The 12 patients who received remnant resections had 10 PDACs and 2 intraductal papillary mucinous carcinomas. The median survival time was 31.6 months, and 8 patients are still alive.

**Conclusions:**

Long-term survivors after curative resection for pancreatic cancer should receive follow-up for remnant pancreatic cancer, and aggressive resection should be considered for more favorable prognosis of PDAC.

## Introduction

Pancreatic cancer is known to have a high frequency of postoperative recurrences and a poor prognosis. Pancreatic cancer is now the fifth leading cause of cancer-related deaths in Japan [1], and most patients develop a recurrence of the same cancer within 1 or 2 years after tumor removal [2]. The most frequent recurrence patterns for pancreatic cancer after resection are local recurrence, hepatic metastasis, and peritoneal dissemination [3–6]. The high frequency of reported recurrences has been attributed to malignant cells remaining on resected margins in up to 50 % of cases even after macroscopically curative resection [7]. This R1-like situation could explain the high local recurrence rate [7]. Another reason is the presence of systemic occult disease at the time of operation in most of the patients, which could lead to distant metastasis in the liver (50 % of resected patients) or peritoneum (25 %) [5, 7–9].

Remnant pancreatic cancer sometimes appears in survivors after curative resection for invasive ductal adenocarcinoma of the pancreas (PDAC). However, there are few reports on the development of pancreatic cancer in the remnant pancreas after pancreatectomy [1], [10]. Consequently, the clinical characteristics of remnant pancreatic cancer after curative resection for PDAC are not well known. The aim of this study was to evaluate clinicopathological findings regarding remnant pancreatic cancer in PDAC survivors.

## Materials and methods

Between 1980 and 2011 at the Department of Gastroenterological Surgery of Tokyo Women’s Medical University, 826 patients who had curative resections for PDAC were studied retrospectively. The remnant pancreatic cancer was detected by means of the serum levels of carcinoembryonic antigen and CA19-9 as well as computed tomography (CT), abdominal ultrasonography, and magnetic resonance imaging (MRI).

The patients were followed up in the outpatient clinic of our hospital or a related hospital and clinic every 3–6 months, and most of the follow-up data collected from these clinical hospital files were studied. (They were evaluated by examination every 3–4 months in the outpatient clinic of our hospital and every 3–6 months in the related hospital and clinic.)

A remnant pancreatic cancer was defined by the following criteria: (1) pathologically negative surgical margins at the first surgery (R0 operation) without M1 but with extra regional lymph nodes according to the UICC. (2) No recurrent tumor detected by CT or MRI examination within 1 year after the first operation. (3) The tumor appeared to develop and exist in the remnant pancreas. Twenty-three patients (2.8 %) fulfilling these criteria were ultimately employed for this retrospective study (Table 1).

**Table 1:** Characteristics of primary pancreatic cancer

Primary operation											
No	Age	Gender (M/F)	Location	Operation	Differentiation	Tumor size(mm)	UICC	Stage	Pancreatic duct margin	Preoperative CA19-9 levels(U/ml)	Postoperative adjuvant chemotherapy
1	67	M	Head	PD	Moderately	25	T3N1M0	IIB	(−)	Not described	(−)
2	66	F	Body	DP	Moderately	30	T2N0M0	IB	(−)	56	(−)
3	54	M	Body	DP	Moderately	17	T3N0M0	IIA	(−)	Not described	(−)
4	53	M	Head	PPPD+PV	Well	15	T3N0M0	IIA	(−)	1	(−)
5	63	M	Head	PPPD	Moderately	13	T1N0M0	IA	(−)	Not described	(−)
6	72	M	Head	PD+PV	Moderately	30	T3N0M0	IIA	dysplasia	Not described	(−)
7	57	M	Head	PD	Moderately	15	T3N1M0	IIB	(−)	19	(−)
8	60	M	Body	DP	Well	33	T3N0M0	IIA	(−)	179	GEM
9	56	M	Head	PPPD	Moderately	45	T3N1M0	IIB	(+)	1910	S-1
10	59	F	Head	PPPD+PV	moderately	37	T3N1M1	IV	(−)	420	GEM
11	55	M	Head	PPPD+PV	Moderately	45	T3N0M0	IIA	(+)	784	GEM
12	58	F	Body	DP	Moderately	30	T3N0M0	IIA	(+)	Not described	(−)
13	82	M	Body	DP+PV	Moderately	20	T3N0M0	IIA	PanIN-1B	8	(−)
14	69	M	Body	DP	Moderately	15	T3N0M0	IIA	(−)	113	(−)
15	59	F	Head	DPPHR	Poorly	15	T3N0M0	IIA	(−)	64	(−)
16	55	F	Body	DP+Ace	Moderately	80	T3N1M1	IV	(−)	362	immunotherapy
17	62	M	Head	PPPD+PV	Moderately	30	T3N1M0	IIB	(−)	138	GEM
18	65	F	Body	DP	Moderately	15	T1N0M0	IA	(−)	15	(−)
19	70	F	Body	Dorsal pancreas resection+DP	Moderately	13	T2N0M0	IB	(−)	14	GEM
20	57	M	Head	PPPD+PV	Moderately	15	T3N1M0	IIB	(−)	19	GEM
21	48	M	Body	DP	Papillary	25	T3N0M0	IIA	(−)	24	GEM+immunotherpy
22	82	F	Body	DP	Moderately	15	T3N1M0	IIB	(−)	9	GEM+immunotherpy
23	63	F	Head	PPPD	Well	20	T3N1M0	IIB	(−)	84	S-1

*PD* pancreaticoduodenectomy, *DP* distal pancreatectomy, *PPPD* pylorus preserving PD, *PV* portal vein resection and reconstruction, *DPPHR* duodenum preserving pancreas head resection, *y* year, *m* month, *IOR* intraoperative radiation, *BSC* best supportive care, *SSPPD* subtotal stomach preserving pancreaticoduodenectomy, *IPMC* intraductal papillary mucinous carcinoma *GEM* gemcitabine

All of these 23 cases were pathologically confirmed by histological information to be PDAC with R0 treatment at the first operation. Clinicopathological features including sex, age, primary and secondary tumor characteristics, and long-term survival data were collected from hospital records. These factors related to the primary operation including sex, age, location, operation, degree of differentiation, tumor size, N of UICC (lymph node state), pancreatic duct margin, preoperative CA19-9 levels, perineural invasion, and postoperative adjuvant chemotherapy were analyzed using both univariate and multivariate analyses.

### Statistical analysis

The survival times of unresected and resected patients were estimated using the Kaplan–Meier method and compared using the log-rank test. A univariate and multivariate Cox proportional hazards model was used to evaluate which factors demonstrated an independent effect on disease-free intervals (DFIs). *P* values less than 0.05 were considered statistically significant. Analysis was performed using SPSS Statistics 22.0 (IBM Corp., Chicago, IL).

## Results

At the first surgery, the tumor was located in the pancreatic head in 12 patients and in the pancreatic body in 11 patients. Pylorus-preserving pancreatoduodenectomy (PPPD) was performed in 8 patients, pancreatoduodenectomy (PD) in 3 patients, duodenum-preserving pancreatic head resection (DPPHR) in 1 patient, and distal pancreatectomy (DP) in 11 patients (Tables 1, 2). A well-differentiated tubular adenocarcinoma was detected in 3 patients, a moderately differentiated tubular adenocarcinoma in 18 patients, a papillary adenocarcinoma in 1 patient, and a poorly differentiated adenocarcinoma in 1 patient. These patients were classified into stages according to UICC classification as follows: 2 in IA, 2 in IB, 10 in IIA, 7 in IIB, and 2 in IV. All patients underwent curative resection. The median and mean DFIs for these patients were 53.6 and 74.2 months (range, 15–240), respectively. Regarding treatment procedures, 12 patients underwent total excision of the remnant pancreas, 6 received chemotherapy, and 5 received the best supportive care (BSC). The six patients treated with chemotherapy consisted of four who received chemotherapy after refusing surgery despite resectable PDAC and two with unresectable PDAC due to locally advanced tumors. The five BSC patients consisted of three with unresectable PDAC (2 with locally advanced tumor and 1 with liver metastasis) and two with resectable PDAC who refused surgery.

**Table 2:** Characteristics of remnant pancreatic cancer

Remnant operation							
No	DFI	Treatment	Differentiation	UICC	Stage	Prognosis(after 2nd operation)	Recurrence form
1	20 years	BSC(resection impossible)	(−)			Death(6 m)	Peritoneum
2	11 year 11 month	Remnant pancreas resection+PV	Moderately	T3N1M0	IIB	Death(11 month)	Peritoneum
3	9 year 5 month	Chemotherapy(resection possible)	(−)			Death(3 year)	Liver, peritoneum
4	10 year 1 month	Chemotherapy(resection possible)	(−)			Death(1 year 4 month)	Liver, peritoneum
5	10 year 2 month	BSC(resection impossible)	(−)			Death(6 month)	Liver, peritoneum
6	9 year 9 month	BSC(resection impossible)	(−)			Death(2 month)	Liver, peritoneum, lung
7	9 year 2 month	Remnant pancreas resection+remnant stomach resection+IOR	Moderately	T3N1M0	IIB	Death(4 month)	Pleura
8	5 year 8 month	Remnant pancreas resection	Well	T1N0M0	IA	Alive(7 year)	(−)
9	6 year 3 month	Remnant pancreas resection	Moderately	T3N0M0	IIA	Alive(1 year 3 month)	(−)
10	4 year 5 month	Chemotherapy(resection possible)	(−)			Death(1 year 6 month)	Liver, peritoneum
11	2 year 5 month	Chemotherapy(resection possible)	(−)			Death(9 month)	Liver, peritoneum
12	2 year 5 month	Remnant pancreas resection	Moderately	T3N0M0	IIA	Death(2 year 1 month)	Liver
13	3 year5 month	BSC(resection possible)	(−)			Death(10 month)	Liver, peritoneum
14	3 year 3 month	Remnant pancreas resection+PV	Well	T3N1M0	IIB	Death(2 year 6 month)	Liver, lung
15	2 year 9 month	Remnant pancreas resection	Poorly	T3N0M0	IIA	Alive(7 year 6 month)	(−)
16	2 year 6 month	Remnant pancreas resection+remnant stomach resection	IPMC	TisN0M0	0	Alive(8 month)	(−)
17	1year 3month	DP	Moderately	T3N1M0	IIB	Alive(3 year)	Peritoneum
18	2 year 7 month	DPPHR	IPMC	TisN0M0	0	Alive(3 year 8 month)	(−)
19	2year 9 month	Chemoradiotherapy(resection impossible)	(−)			Death(12 month)	Peritoneum
20	2 year 8 month	Remnant pancreas resection	Moderately	T3N1M0	IIB	Alive(6 month)	(−)
21	13 year 1 month	Chemoradiotherapy(resection impossible)	(−)			Dead(4 year 5 month)	Peritoneum
22	1 year 6 month	BSC(resection possible)	(−)			Dead(2 year 2 month)	Peritoneum
23	5 year 2 month	Remnant pancreas resection	Moderately	T3N1M0	IIB	Alive(1 month)	(−)

*DFI* disease free interval *PD* pancreaticoduodenectomy, *DP* distal pancreatectomy, *PPPD* pylorus preserving PD, *PV* portal vein resection and reconstruction, *DPPHR* duodenum preserving pancreas head resection, *y* year, *m* month, *IOR* intraoperative radiation, *BSC* best supportive care, *SSPPD* subtotal stomach preserving pancreaticoduodenectomy, *IPMC* intraductal papillary mucinous carcinoma *IOR* intraoperative radiation therapy

**Fig. 1:**
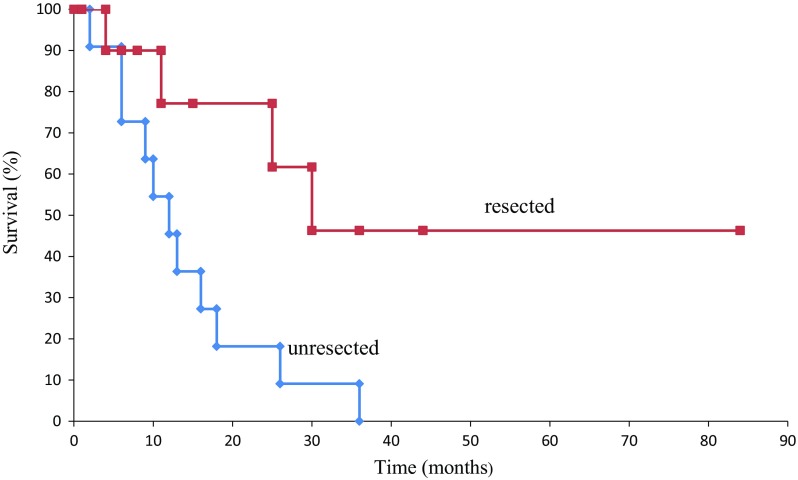
Kaplan-Meier curve for overall survival between resected and unresected patients for the remnant pancreatic cancer

The pathological findings of the remnant pancreas in the 12 resected cases were PDAC in 10 patients and intraductal papillary mucinous carcinoma with an associated invasive carcinoma in 2 patients. The 12 patients were found to be in the following stages (UICC classification): 2 in 0, 1 in IA, 3 in IIA, and 6 in IIB. The same histopathological features in specimens from the first and second operations were recognized in 8 of the 12 (67 %) cases. The mortality and morbidity rates of resected patients were 0 and 41.6 %, respectively. Complications were observed in five patients: 2 with delayed gastric emptying, 2 with intra-abdominal abscess, and 1 with sepsis. The average length of hospital stay was 34 days (13–70). No unresected patients had survived, and eight patients who underwent total excision of the remnant pancreas survived. The 5-year survival rate for all the 826 studied resected pancreatic cancer patients was 21.3 %. The mean and median survival times after the second operation were 23.7 (range, 1–90) and 31.6 months, respectively. The 1-year and 3-year survival rates and the median survival time were 45.5, 9.1 %, and 12 months for unresected patients, respectively, contrasting with 79.6, 53 %, and not obtained for resected patients, respectively (*P* = 0.0049 by log-rank test) (Fig. 1).

Recurrences were observed in 16 patients (13 with peritoneal dissemination [56.5 %], 9 with liver metastasis [39.1 %], 2 with lung metastasis [8.7 %], and 1 with pleural metastasis [4.3 %]). A univariate analysis for DFI showed no significant difference by any of clinicopathological features. None of the lymph node state, degree of differentiation, and perineural invasion could predict survival. Therefore, multivariate analysis for DFI could not have been investigated.

## Discussion

The 5-year survival rate reported after surgical resection of PDAC is approximately 20 % [11]. Most patients develop recurrence within 1 or 2 years after tumor removal [3, 12, 13]. The surgical mortality rate associated with pancreatectomy has decreased to less than 5 % despite high morbidity rates and recent improvements in operative technique and perioperative management that have resulted in an increase in the number of long-term survivors after pancreatectomy [14–20]. Thus, there have been some recent reports regarding cases of remnant pancreatic cancer after pancreatectomy [21–24]. We evaluated clinicopathological findings of the remnant pancreatic cancer in survivors of PDAC.

It is difficult to define whether a pancreatic carcinoma developing in the remnant pancreas after a pancreatectomy for PDA is a local recurrence or a newly developed primary cancer [2]. The remnant pancreatic cancer was defined in this study according to those criteria mentioned earlier. In the literature, the time interval from primary PDA to remnant pancreatic cancer ranges from 12 to 89 months (mean 37.6 months), using our definition (Table 3) [1, 2, 21–30]. On the other hand, the time interval in our studied cases ranged from 15 to 240 months (mean 74.2 months) (Table 2), which is much longer than the previous reports. This result suggests that long-term follow-up after surgery for PDAC may be needed even beyond 5 years to monitor for remnant pancreatic cancer. In Japan, postoperative follow-up of patients with resected pancreatic cancer by means of CT or MRI examination every 3–6 months is generally warranted and covered by insurance. Because most patients with resected pancreatic cancer are likely to develop recurrence soon after resection, an intensive follow-up schedule is considered necessary to find recurrence in a remnant pancreas. Whether such intensive follow-up could be performed for the long term may be debatable when considering cost-effectiveness. The results of this study indicated that a newly developed cancer in the remnant pancreas could be detected by performing CT or MRI examination every 6–12 months for patients having survived for 5 years. Such long-term survivors could be few, but it is important to implement intensive follow-up beyond 5 years for such patients.

**Table 3:** Reported cases of resected remnant pancreatic cancer

First operation								Remnant pancreeatic cancer							
No	Age	Gender (M/F)	Location	Operation	Differentiation	UICC	Stage	Term to diagnosis	Treatment	Differentiation	UICC	Stage	Prognosis	Recurrence form	Author
1	52	F	Head	PPPD	Pap	T3N1M0	IIB	1 year10 month	DP+GR	Pap	Not described		Not described	(−)	Wada [24]
2	67	F	Body/tail	DP	Well	T1N0M0	IA	7 year4 month	PPPD	Moderately	T1N1M0	IB	Alive(8 month)	(−)	Eriguchi [25]
3	58	M	Head	PPPD	Pap/well	T2N0M0	IB	3 year	DP	Pap	T2N0M0	IB	Alive(6 year3 month)	Liver	Tajima [2]
4	63	M	Head	PD	Well to moderately	T3N0M0	IIA	3 year7 m	DP	Well	not described		Alive(10 m)	(−)	Takamatsu [22]
5	65	M	Head	PPPD	Well	T1N0M0	IA	7 year	DP	Well	T3N1M0	IIB	Alive(10 m)	(−)	Koizumi [26]
6	67	M	Body	DP	Well	T1N0M0	IA	2 year9 month	PPPD	Moderate to poorly	T3N1M0	IIB	Alive(8 month)	Liver	Koizumi
7	63	M	Head	PD+PV	Moderately	T3N0MX	IIA	1 year	DP+GR+C(T)R	Moderately	not described		Alive(2 year)	LN	Valle [21]
8	60	M	Body	DP	Moderately	T3N0M0	IIA	2 year2 month	PPPD	Poorly	T2N0M0	IB	Dead(7 month)	peritoneum	Doi [27]
9	44	M	Head	PPPD	Not described	T2N1MX	IIB	3 year 4 month	DP	Not described	T2NXM0		Alive(22 month)	(−)	D’Amato [28]
10	72	F	Head	PPPD	Moderately	T3N0M0	IIA	2 year5 month	DP+lateral segmentectomy	Moderately	not described		Dead(5 month)	Liver	Miura [23]
11	52	F	Head	PPPD	Pap	T3N1M0	IIB	1y10 month	DP	Pap	not described		Dead(44 month)	Not described	Miura
12	67	M	Head	PD	Well	T2N1M0	IIB	5y8 month	DP	Well	not described		Alive(2 month)	(−)	Kinoshita [29]
13	55	F	Head	SSPPD+PV	Anaplastic	T1N0M0	IA	2 year	DP	Well	T2N0M0	IB	Alive(5 year4 8 month)	(−)	Hashimoto [1]
14	69	F	Head	SSPPD	Pap	T1N0M0	IA	3 year 2 mmonth	DP	Well	T1N1M0	IB	Alive(10 month)	(−)	Hashimoto
15	80	M	Head	PPPD	Moderately	T3N0M0	IIA	1 year 9 month	DP	Moderately	T3N1M0	IIB	Dead(1 year 6 month)	Not described	Hashimoto
16	60	M	Tail	DP	Pap	T2N1M0	IIB	2 year 9 month	TP	Pap	T1N0M0	IA	Alive(7 year 1 month)	(−)	Hashimoto
17	75	F	Tail	DP	Well	T3N1M0	IIB	3 year 3 month	TP	Well	T1N0M0	IA	Alive(1 year 1 month)	(−)	Hashimoto
18	76	M	Body	DP	Moderately	T1N0M0	IA	1 year 11 month	TP	Moderately	T3N1M0	IIB	Dead(1 year 1 month)	(−)	Hashimoto
19	62	F	Head	PPPD+PV	Well	T3N1M0	IIB	1 year 5 month	chemotherapy	Not described	not described		Dead(11 month)	Liver	Hashimoto
20	66	M	Head	PD	Moderately	T3N0M0	IIA	1y4 m	TP	Moderately	T3N0M0	IIA	Alive(61 month)	(−)	Miyazaki [30]
21	68	F	Head	PD+PV	Moderately	T3N1M0	IIB	3 year 5 month	TP	Moderately	T3N0M0	IIA	Alive(61 month)	(−)	Miyazaki
22	62	F	Head	PD+PV	Moderately	T3N1M0	IIB	2 year 8 month	TP	Moderately	T3N1M0	IIB	Dead(15 month)	Peritoneum	Miyazaki
23	76	F	Head	PD	Well	T3N0M0	IIA	3 year 5 month	TP	Moderately	T3N1M0	IIB	Dead(25 month)	Peritoneum,liver	Miyazaki
24	80	M	Body	DP	Moderately	T3N0M0	IIA	2 year	TP	Well	T3N0M0	IIA	Alive(28 monthm)	(−)	Miyazaki
25	67	M	Head	PD	Moderately	T2N0M0	IB	7 year 5 month	TP	Poorly	T1N0M0	IA	Alive(18 month)	(−)	Miyazaki

*PD* pancreaticoduodenectomy, *DP* distal pancreatectomy, *PPPD* pylorus preserving PD, *SSPPD* subtotal stomach preserving pancreaticoduodenectomy, *PV* portal vein resection and reconstruction, *y* year, *m* month, *GR* distal gastrectomy, *C(T)R* toransvers colon resection

The same pathological differentiation of tumors between primary and second operation in our study was recognized in 8 (67 %) of the 12 resected cases, consistent with a previous report indicating 16 (69.6 %) of the 23 described cases as listed in Table 2. Launois et al. [31] observed 32 % of multifocal carcinomas of the pancreas in a series of 47 total pancreatectomies for patients with PDAC. Likewise, a considerably high incidence of multicentric precancerous foci in the pancreas has been documented in patients with PDAC [2]. In PDAC, multicentric lesions were found in 16–34 % of cases [32–35], implying that a minute cancerous focus is likely to exist in the remnant pancreas at the time of the initial surgery [22].

These results suggest that multicentric metachronous pancreatic cancers may develop in the remnant pancreas. Kleff et al. [10] reported that 7 (3.1 %) of the 227 patients who underwent initial pancreatic resection for pancreatic cancer developed remnant pancreatic cancer, consistent with the incidence of remnant pancreatic cancer in our results. Therefore, multicentric foci of PDAC may develop metachronously not only after a short interval but also after a longer interval as a new primary cancer.

Miyazaki et al. [30]. reported that repetitive pancreatectomy may be beneficial for the prognosis in selected patients with isolated local recurrence in the remnant pancreas after primary pancreatectomy for pancreatic cancer without increased operative morbidity or mortality. They recognized 11 of 67 patients with isolated local recurrences only in the remnant pancreas who underwent repetitive pancreatectomies. Moreover, at the primary operation, 6 (67 %) of the 9 patients with R0 resection had the same pathological features as in our study.

In this study, we showed that patients with remnant pancreatic cancers who were resected had a better prognosis than those that were unresected. This suggests that remnant pancreatectomy for pancreatic cancer is feasible and may prolong survival. Although early discovery of remnant pancreatic cancer would be difficult, adequate interval follow-up with imaging examinations is important. Further studies are required to elucidate the carcinogenic mechanism in the remnant pancreas.

In conclusion, survivors after curative resection for pancreatic cancer should receive follow-up for the remnant pancreas for an extended period. Aggressive resection of the remnant pancreas should be considered for a more favorable prognosis of patients with PDAC.
